# Metabolic perturbations associated with the consumption of a ketogenic medium-chain TAG diet in dogs with idiopathic epilepsy

**DOI:** 10.1017/S0007114518001617

**Published:** 2018-07-13

**Authors:** Tsz Hong Law, Holger A. Volk, Yuanlong Pan, Brian Zanghi, Elizabeth J. Want

**Affiliations:** 1 Department of Clinical Science and Services, Royal Veterinary College, Hatfield AL9 7TA, UK; 2 Division of Integrative Systems Medicine and Digestive Disease, Imperial College, London SW7 2AZ, UK; 3 Nestlé Purina Research, One Checkerboard Square, St Louis, MO 63164, USA

**Keywords:** Ketogenic diets, Medium-chain TAG, Metabolic profiling, Odd-chain fatty acids

## Abstract

Consumption of diets containing medium-chain TAG (MCT) has been shown to confer neuroprotective effects. We aim to identify the global metabolic perturbations associated with consumption of a ketogenic diet (medium-chain TAG diet (MCTD)) in dogs with idiopathic epilepsy. We used ultra-performance liquid chromatography-MS (UPLC-MS) to generate metabolic and lipidomic profiles of fasted canine serum and made comparisons between the MCTD and standardised placebo diet phases. We identified metabolites that differed significantly between diet phases using metabolite fragmentation profiles generated by tandem MS (UPLC–MS/MS). Consumption of the MCTD resulted in significant differences in serum metabolic profiles when compared with the placebo diet, where sixteen altered lipid metabolites were identified. Consumption of the MCTD resulted in reduced abundances of palmitoylcarnitine, octadecenoylcarnitine, stearoylcarnitine and significant changes, both reduced and increased abundances, of phosphatidylcholine (PC) metabolites. There was a significant increase in abundance of the saturated C17 : 0 fatty acyl moieties during the MCTD phase. Lysophosphatidylcholine (17 : 0) (*P*=0·01) and PC (17:0/20:4) (*P*=0·03) were both significantly higher in abundance during the MCTD. The data presented in this study highlight global changes in lipid metabolism, and, of particular interest, in the C17 : 0 moieties, as a result of MCT consumption. Elucidating the global metabolic response of MCT consumption will not only improve the administration of current ketogenic diets for neurological disease models but also provides new avenues for research to develop better diet therapies with improved neuroprotective efficacies. Future studies should clarify the involvement and importance of C17 : 0 moieties in endogenous MCT metabolic pathways.

The initial use of ketogenic diets (KD) for epilepsy in humans was in the 1920s, in order to mimic the metabolic state and biochemical changes associated with fasting, as fasting was shown to possess anticonvulsant properties^(^ ^1^ ^)^. A plethora of anecdotal reports and published literature have since highlighted the use of different diet therapies and KD for epilepsy, all showing varying levels of anticonvulsant properties in humans^(^ ^2^ ^,^ ^3^ ^)^. Canine epilepsy is thought to be similar in pathology, heterogeneity, aetiology and clinical manifestations to its human counterpart, and thus acts as a good translational model^(^ ^4^ ^)^. It has therefore been proposed that KD may also act as an alternative treatment strategy for canine epilepsy^(^ ^5^ ^,^ ^6^ ^)^. A novel KD (medium-chain TAG diet (MCTD)) developed for canine consumption, with relatively low fat and medium-chain TAG (MCT) levels, was recently shown to significantly reduce both seizure frequency and the number of days with seizure occurrence in dogs with idiopathic epilepsy^(^ ^7^ ^)^. This MCTD contained caprylic (8 : 0), capric (10 : 0) and lauric (12 : 0) SFA^(^ ^7^ ^)^, which is in accordance with the MCT content present in other MCT KD^(^ ^8^ ^,^ ^9^ ^)^. The use of MCT KD transcends beyond purely anticonvulsant purposes and has been shown to improve behaviour in dogs^(^ ^10^ ^)^. Importantly, it has also been shown to enhance cognitive functioning not only in humans diagnosed with epilepsy^(^ ^11^ ^)^ but also for patients with Alzheimer’s disease and type 1 diabetes^(^ ^12^ ^,^ ^13^ ^)^. Furthermore, beyond neurological properties, medium-chain fatty acids (MCFA) have also shown positive effects in obesity and cancer-related disease models in humans^(^ ^14^ ^–^ ^16^ ^)^.

With regard to the anticonvulsant effects of MCFA, published literature thus far supports the hypothesis that caprylic, capric and lauric FA, the main constituents within the MCT KD, possess direct mechanistic effects^(^ ^8^ ^,^ ^9^ ^,^ ^17^ ^)^. An *ex vivo* rat hippocampal slice model of epileptiform activity showed that capric acid, acting as a non-competitive antagonist, binds to *α*-3-hydroxy-5-methyl-4-isoxazolepropionic acid receptors, which exerts inhibitory effects on excitatory neurotransmission^(^ ^17^ ^)^. Research endeavours focusing on *in vitro* and mouse models of MCT in epilepsy have also highlighted anticonvulsant effects of specific branched-chain fatty acids, such as the C9 (4-methyloctanoic), C10 (4-methylnonanoic) and C12 (2-butyloctanoic) fatty acids (FA)^(^ ^9^ ^,^ ^17^ ^,^ ^18^ ^)^. However, it is unknown whether these potentially related metabolites are in fact implicated in the anticonvulsant effectiveness of MCT KD in an *in vivo* model of epilepsy. Others have suggested that MCFA indeed play a less direct role and hypothesised that MCFA exert downstream effects, by way of plasma albumin binding site metabolite displacement, on tryptophan metabolism, which in turn increases seizure thresholds^(^ ^19^ ^–^ ^21^ ^)^.

The global and likely multiparametric metabolic response associated with MCT and/or MCFA consumption remains unclear. Using research techniques, such as metabolic profiling, to fully characterise the global metabolic response of medium-chain lipid consumption is a crucial initial step in elucidating diet-induced neurological effects of MCFA, which are likely to be variable and multifactorial^(^ ^22^ ^)^. Metabolic profiling research techniques, also commonly known as ‘metabolomics’ or ‘metabonomics’, incorporate the measurement of a ‘multiparametric metabolic response of living systems to pathophysiological, genetic or environmental stimuli’^(^ ^23^ ^)^. Ultra-performance liquid chromatography coupled to MS (UPLC-MS) is an analytical technique commonly used in untargeted metabolic profiling studies. Although a biological sample can be directly infused into a mass spectrometer, a chromatographic step is typically used before mass analysis^(^ ^24^ ^)^. Chromatography facilitates the separation of metabolites within biological samples on the basis of their intrinsic metabolic properties, which further improves the quality of downstream mass analysis of metabolites present^(^ ^24^ ^)^. Furthermore, different chromatographic techniques can be used to facilitate improved separation of a specific metabolite class, which may be present in a biological sample, such as lipid profiling (LP) chromatography, which facilitates the separation and detection of lipids^(^ ^25^ ^)^. Elucidating the pathways of MCT metabolism and the global metabolic response of MCT consumption in canine epilepsy will provide insights into how MCT may confer neurological effects^(^ ^7^ ^)^. The primary aim of this study was therefore to determine the global metabolic response of ketogenic MCTD consumption on serum biofluid metabolic profiles in dogs with idiopathic epilepsy.

## Methods

This study was conducted in accordance with the guidelines laid down in the International Cooperation on Harmonization of Technical Requirements for Registration of Veterinary Medical Products GL9 Good Clinical Practices (GCP) and the European Agency for the Evaluation of Medical Products (EMEA). The study protocol was approved by the local Ethics and Welfare Group (EWG) (URN 2011 1132). The 6-month, randomised, double-blinded, placebo-controlled, cross-over, novel medium-chain TAG diet trial study for dogs with idiopathic epilepsy has previously been reported^(^ ^7^ ^)^. Briefly, the study population consisted of twenty-one dogs of seventeen different breeds detailed in the online Supplementary Table S1. The study population consisted of fifteen males, of which ten were neutered and five were intact, and six females, of which four were neutered and two were intact (online Supplementary Table S1). The dogs had a mean age of 4·59 (sd 1·73) years and weighed a mean of 29·79 (sd 14·73) kg at the start of the trial (online Supplementary Table S1). The experimental placebo and MCTD formulas were dry extruded kibble (Nestle Purina PetCare) formulated to contain <10 % moisture, at least 28 % crude protein, at least 15 % crude fat and 50 % carbohydrates, with <2 % as crude fibre^(^ ^7^ ^)^. The only composition difference is that zero MCT were added to the placebo formula, and lard was used as fat substitute to ensure that the formulas were isoenergetic (1561 kJ/100 g (373 kcal)/100 g), whereas the test formula contained 5·5 % MCT. MCT content was about 10 % of total formula calories (based on fat as 35·5 kJ (8·5 kcal)/g and MCT as 28·4 kJ/100 g (6·8 kcal)/g). Proximate analysis of both formulas indicated that they were of similar composition, with the exception of MCT, as the placebo diet was void of C12, C10 and C8 FA (each <0·100 % of placebo formula). In this study, canine serum samples were analysed using an untargeted UPLC-MS metabolic profiling approach. The reversed-phase (RP) chromatography facilitated the separation of non-polar /moderately polar molecules, whereas the LP chromatography facilitated the separation of lipids^(^ ^24^ ^,^ ^25^ ^)^.

### Serum sample collection and preparation

Blood samples were collected from the dogs over the duration of the study at visit two on day 90 (sd 2) d and at visit three on day 180 (sd 2) d, corresponding to either placebo diet phase or MCTD phase samples. This study consisted of a cross-over placebo controlled diet trial design where the first diet initiated, either placebo or MCTD, was randomised. Blood was collected after overnight fasting before consumption of respective diets and routine concomitant antiepileptic drugs (AED) the next day. Fasted samples were used in this study in order to interrogate the global shifts in metabolism rather than immediate/short-term changes associated with diet consumption. Further information regarding the collection of canine serum samples can be found in the online Supplementary Table S2. Canine serum was combined with pre-chilled methanol to facilitate protein precipitation. Proteins were removed by centrifugation where the supernatants were subsequently dried by centrifugal evaporation. Dried metabolite extracts were re-suspended in UPLC-grade water for RP chromatography and in methanol–water (1:1) for LP chromatography. Pooled quality control (QC) samples were made using 10 μl of each study sample^(^ ^26^ ^)^. Further information regarding the preparation of canine serum samples can be found in the online Supplementary Table S2.

### Canine serum ultra-performance liquid chromatography-MS data acquisition and extraction

Ultra-performance liquid chromatography separation was performed using an Acquity UPLC system (Waters Corporation). A high strength silica column was used for RP liquid chromatography and a charged surface hybrid column was used for LP liquid chromatography (online Supplementary Table S3). MS was carried out on a Xevo G2-S Q-TOF (Waters MS technologies) mass spectrometer for RP experiments and on a Q-TOF Premier (Waters MS technologies) mass spectrometer for LP experiments (online Supplementary Table S4). Data extraction was performed by peak picking and grouping using the XCMS package in R programming language (open-source software R, version 3.3.2) (online Supplementary Table S5). A QC filtering protocol was used in all UPLC-MS analyses^(^ ^26^ ^)^. Briefly, pooled QC samples were injected ten times before UPLC-MS analysis of study samples to facilitate the conditioning of the chromatographic column. QC samples were subsequently injected approximately once every ten study samples analysed and at the end of the study. A CV of ≥30 % within QC for all extracted metabolite features formed the major criteria for removal of metabolite features that were considered unreliable. Furthermore, metabolite features that were not present in (QCn-1) QC samples were also considered unreliable and removed from further data analysis. The resultant metabolite features list, classified as reliable metabolic features passing the QC filtering protocols, was normalised by median fold change using an in-house normalisation script executed in the R programming language (open source software, R, version 3.3.2).

### Statistical analysis

Patterson *et al*.^(^ ^27^ ^)^ performed power calculations using the results that were acquired from their study, which showed that twenty-two dogs in each group would be sufficient to show significant differences between diet groups using seizure frequency as the major outcome variable. We report only twenty-one dogs, as one dog was excluded owing to an error in the diet dispensed, which came to our notice only during data analysis after completion of the study^(^ ^7^ ^)^. Statistical analysis of canine serum data included paired Student’s *t* test analysis with Benjamini–Hochberg (BH) false discovery rate (FDR) multiple *t* test *P* value correction comparing metabolite intensities between diet groups^(^ ^28^ ^)^. An FDR multiple test correction method is commonly applied in metabolomic studies owing to the large number of metabolite features that are detected and analysed simultaneously. The BH critical value was calculated by (*i*/*m*)*Q*, where *i* is the relative rank of a *P*-value generated from a single *t* test, *m* is the total number of statistical tests calculated and *Q* is the pre-determined FDR (*Q*=0·05). The largest statistical test *P* value result that is *P*<(*i*/*m*)*Q* is considered significant, with all subsequent *P*-values that are smaller also considered significant and adjusted accordingly^(^ ^28^ ^)^. Significant metabolite features (*P*<0·05) were identified by UPLC-tandem MS (UPLC-MS/MS) analysis. Subsequent UPLC-MS/MS verification required a m/z (mass-to-charge) match of ±0·01 *m*/*z* and a retention time match of ±0·5 min to the original UPLC-MS-detected metabolite feature to be considered reliable. Metabolite identification was facilitated by mass spectra fragmentation patterns and matching m/z ratio to metabolites found in online databases, including LIPID MAPS (http://www.lipidmaps.org), HMDB (http://www.hmdb.ca/), METLIN (https://metlin.scripps.edu/index.php) and other published literature^(^ ^29^ ^)^. Relative metabolite abundance fold change was calculated on the basis of the average fold changes for individual paired samples and was relative to the lower-abundance diet phase group.

## Results

**Fig. 1:**
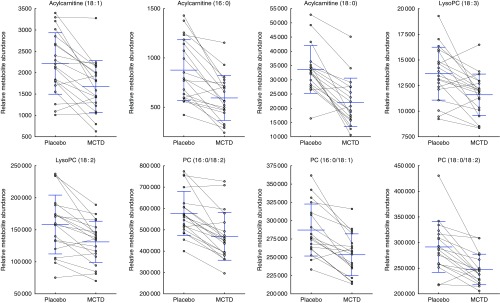
Metabolites detected by ultra-performance liquid chromatography-MS (UPLC-MS) in fasted canine serum, which were significantly higher in abundance during the placebo diet phase when compared with the medium-chain TAG diet (MCTD). Relative metabolite intensities are presented as scatter dot plots with means and standard deviations, where paired samples are highlighted with the connecting line. PC, phosphatidylcholine; LysoPC, lysophosphatidylcholine.

**Fig. 2:**
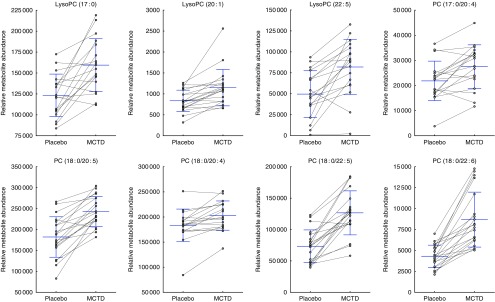
Metabolites detected by ultra-performance liquid chromatography-MS (UPLC-MS) in fasted canine serum, which were significantly higher in abundance during the medium-chain TAG diet (MCTD) when compared with the placebo diet phase. Relative metabolite intensities are presented as scatter dot plots with means and standard deviations, where paired samples are highlighted with the connecting line. PC, phosphatidylcholine; LysoPC, lysophosphatidylcholine.

Consumption of the MCTD resulted in significant changes to the fasted canine serum metabolic profiles when compared with the standardised placebo diet, which contained zero MCT. In all, sixteen metabolites, detected using UPLC-MS/MS techniques, were altered with statistical significance between placebo diet and MCTD phases (Table 1). All metabolites were identified based on UPLC-MS/MS fragmentation experiments and belong to the classes of compounds known as phosphatidylcholine (PC) and acylcarnitine metabolites; examples of these metabolites can be found in the online Supplementary Fig. S1. Palmitoylcarnitine (C16 : 0), octadecenoylcarnitine (C18 : 1), stearoylcarnitine (C18 : 0), LysoPC(18 : 2), LysoPC (18 : 3), PC (16 : 0/18 : 2), PC (16 : 0/18:1) and PC (18 : 0/18 : 2) were shown to be higher in abundance during the placebo diet phase (Fig. 1). Conversely, LysoPC (17 : 0), LysoPC (20 : 1), LysoPC (22 : 5), PC (17 : 0/20 : 4), PC (18 : 0/20 : 5), PC (18 : 0/20 : 4), PC (18 : 0/22 : 6) and PC (18 : 0/22 : 5) were shown to be higher in abundance during the MCTD phase (Fig. 2).

**Table 1:** Metabolites shown to be significantly different in abundance between placebo diet and medium-chanin TAG diet (MCTD) phases*

Metabolite names	Molecular formula	M *v*. P (*P*)	FDR (M *v*. P) (*P*)	LC protocol (ESI mode)	Adduct (detected)	Mass *m*/*z* (detected)	Mass *m*/*z* (theoretical)	Δppm	Fold change	CV (%)
Metabolites higher in abundance during the placebo diet phase										
Acylcarnitine (16 : 0)	C_23_H_45_NO_4_	<0·001	<0·001	LP (+)	[Met+H]	400·3432	400·3421	3	1·62	17
Acylcarnitine (18 : 1)	C_25_H_47_NO_4_	<0·001	0·005	LP (+)	[Met+H]	426·3585	426·3578	2	1·68	14
Acylcarnitine (18 : 0)	C_25_H_49_NO_4_	<0·001	0·005	LP (+)	[Met+H]	428·3736	428·3734	0	1·43	8
LysoPC (18 : 3)	C_26_H_48_NO_7_P	0·002	0·02	LP (+)	[Met+H]	518·3243	518·3241	0	1·20	19
LysoPC (18 : 2)	C_26_H_50_NO_7_P	<0·001	0·005	LP (+)	[Met+H]	520·3406	520·3393	2	1·22	20
PC (16 : 0/18 : 2)	C_42_H_80_NO_8_P	<0·001	<0·001	LP (+)	[Met+H]	758·5724	758·5694	4	1·24	13
PC (16 : 0/18 : 1)	C_42_H_82_NO_8_P	<0·001	0·003	RP (−)	[Met+FA-H]	804·5747	804·5760	2	1·14	2
PC (18 : 0/18 : 2)	C_44_H_84_NO_8_P	<0·001	0·012	RP (−)	[Met+FA-H]	830·5904	830·5917	2	1·18	3
Metabolites higher in abundance during the MCTD phase										
LysoPC (17 : 0)	C_25_H_52_NO_7_P	<0·001	0·013	RP (−)	[Met+FA-H]	554·3457	554·3463	1	1·33	4
LysoPC (20 : 1)	C_28_H_56_NO_7_P	<0·001	0·006	LP (+)	[Met+H]	550·3893	550·3867	5	1·43	17
LysoPC (22 : 5)	C_30_H_52_NO_7_P	<0·001	0·012	RP (−)	[Met+FA-H]	614·3457	614·3462	1	2·62	6
PC (17 : 0/20 : 4)	C_45_H_82_NO_8_P	0·002	0·032	RP (−)	[Met+FA-H]	840·5754	840·5760	1	1·39	2
PC (18 : 0/20 : 5)	C_46_H_82_NO_8_P	<0·001	<0·001	LP (+)	[Met+H]	808·5895	808·5851	5	1·44	11
PC (18 : 0/20 : 4)	C_46_H_84_NO_8_P	<0·001	0·019	RP (−)	[Met+FA-H]	854·5901	854·5917	2	1·13	3
PC (18 : 0/22 : 6)	C_48_H_84_NO_8_P	<0·001	<0·001	LP (+)	[Met+H]	834·6078	834·6007	8	2·15	14
PC (18 : 0/22 : 5)	C_48_H_86_NO_8_P	<0·001	<0·001	LP (+)	[Met+H]	836·6215	836·6164	6	1·90	16

FDR, false discover rate; M, MCTD phase; P, placebo diet phase; ESI, electrospray ionisation; *m*/*z*, mass:charge ratio; Δppm, deviation between measured mass and theoretical mass in ppm calculated; CV (%), CV calculated based on total number of pooled quality control samples acquired during experimental run; *P* value correction; LP, lipid profiling; Met, metabolite; PC, phosphocholine; RP, reversed phase; FA, formate; UPLC–MS, ultra-performance liquid chromatography-MS. *Metabolites were detected in ESI+ and ESI− modes using LP-UPLC-MS and RP-UPLC-MS (percentages). Significant metabolites were determined using paired Student’s *t* test with FDR *P* value correction (*P* < 0·05) and identified in UPLC–MS/MS experiments, respectively.

## Discussion

It is interesting that the fundamental difference in diet composition between the MCTD and placebo diet was the presence or absence of MCT, and that this difference was not directly apparent between the metabolic profiles. As fasted serum samples were used in this study to interrogate the global shifts in metabolism associated with diet consumption, the lack of MCT and MCFA detection may be attributed to the natural metabolic rate of these metabolites. MCT, as opposed to long-chain TAG (LCT), are used to a greater and more efficient degree^30^ ^,^ ^31^. The transport process from intestinal lumen to the liver for MCT, in comparison with LCT, is much faster and more efficient^(^ ^30^ ^,^ ^31^ ^)^. Furthermore, transport of MCFA into the mitochondrial matrix for *β*-oxidation does not require the carnitine shuttle system that is necessary for long-chain FA (LCFA)^(^ ^30^ ^)^. MCT are therefore metabolised at a quicker rate when compared with LCT. Furthermore, all dogs included in this study were chronically treated with the AED phenobarbital (PB). It is thought that PB, primarily metabolised in the liver, also promotes induction of the cytochrome-P450 (CYP) enzyme system^(^ ^32^ ^)^. As it has been shown that MCT are metabolised by some CYP4 isoforms, the dogs in this study, all chronically treated with PB, may have had increased rates of MCT metabolism^(^ ^33^ ^)^. Unfortunately, it is not yet entirely clear whether the exact CYP enzymes involved in their metabolism are the same. Considering the rate at which MCT are normally metabolised compared with LCT, even if MCT and/or MCFA metabolites were initially detected in the fasted serum samples, their intensities may have been too low to either pass metabolite feature QC filtering protocols applied in this study or to be considered significant in subsequent statistical tests.

When considering global shifts in lipid metabolism, as a result of MCTD consumption, MCT and/or MCFA metabolites may have been metabolised into downstream products by *de novo* hepatic synthesis and/or lipogenesis pathways (online Supplementary Fig. S2). In a rat hepatocyte model study, C^14^-radiolabelled lauric FA (C12 : 0) was shown to be rapidly converted to palmitic FA (C16 : 0) by two successive elongations^(^ ^31^ ^)^. In another randomised cross-over study, over-feeding of an MCT-enriched diet also resulted in elevation of serum C16 : 0, C18 : 0 and C18 : 1 FA^(^ ^34^ ^)^. Hill *et al.* ^34^ hypothesised that it was highly likely that excess dietary MCT results in *de novo* hepatic synthesis of LCFA by chain elongation and/or desaturation of MCFA. Others have since shown that MCT consumption not only increases MCFA in portal circulation but also C16 : 0, C18 : 0, C18 : 1 and C20 : 4 FA moieties in the lymphatic TAG^(^ ^35^ ^)^. It was shown that levels of linoleic acid (C18 : 2) in lymphatic TAG correlated with the type of TAG consumed, with more C18 : 2 moieties seen in LCT consumption when compared with MCT consumption^(^ ^35^ ^)^. Results presented by You *et al.* ^(34)^ and Hill *et al.* ^(35)^ are in accordance with the results presented in this study, whereby the diet containing relatively higher levels of LCT resulted in higher abundance of C18 : 2 moieties, and diets containing MCT resulted in higher abundances of C18 : 0 and C20 : 4 moieties. Therefore, we speculate that MCT may have been metabolised, by way of *de novo* lipogenesis, potentially into fatty acyl moieties shown to be significantly higher in abundance during the MCTD, such as PC metabolites containing C18 : 0 fatty acyl moieties (online Supplementary Fig. S2).

Consumption of the MCTD, when compared with the standardised placebo diet, resulted in significant differences in serum metabolites, predominantly PC metabolites. Most interestingly, lysoPC (17:0) and PC (17:0/20:4) were higher in abundance during the MCTD phase. Fatty acid metabolism typically involves multiple rounds of 2-C cleavage from FA by *β*-oxidation to produce acetyl-CoA, which can be used in other metabolic pathways such as the Krebs cycle, and the acyl-CoA molecule where the FA moiety becomes two carbons shorter (online Supplementary Fig. S3). Therefore, under normal biological pathways, it is uncommon to see FA or PC with an odd number of fatty acyl moieties. Previously, it was thought that odd-chain FA, such as C15 : 0 or C17 : 0, were only introduced into the body through dietary consumption, predominantly from ruminant fats^(^ ^36^ ^)^. However, recently it was shown that some odd-chain FA, such as C17 : 0, are substantially endogenously biosynthesised, whereas others, such as C15 : 0, are indeed introduced through dietary intake^(^ ^37^ ^)^. Although the exact mechanisms of C17 : 0 biosynthesis are unknown, it has been hypothesised that propionyl-CoA elongation and/or *α*-oxidation of C18 : 0 contributed to C17 : 0 FA biosynthesis^(^ ^37^ ^)^. In lipid metabolism, *α*-oxidation refers to a metabolic process that results in the removal of a single carbon unit from a fatty acyl chain, whereas *β*-oxidation refers to the metabolic removal of two carbon units. Jenkins *et al.* ^(37)^ showed that phytanic acid, a substrate for *α*-oxidation, significantly decreased C17 : 0 biosynthesis by competitive inhibition, further suggesting that C17 : 0 is biosynthesised by *α*-oxidation. Here we present data showing significant increases in C17 : 0 moieties during the MCTD phase when compared with the placebo, where the only difference between the diet phases was the presence or absence of medium-chain (C8, C10 and C12) fatty acyl moieties. This further suggests that C17 : 0 may indeed be biosynthesised via lipogenesis processes (online Supplementary Fig. S2). Furthermore, we also showed higher abundances of PC metabolites containing C18 : 0 moieties during the MCTD phase, further allowing the potential for *α*-oxidative biosynthesis of C17 : 0 from C18 : 0 moieties, as suggested by Jenkins *et al.* ^(^ ^37^ ^)^.

Detection of C17 : 0 moieties in lipid metabolites is particularly interesting when considering the neuroprotective properties of triheptanoin, a TAG consisting of three heptanoate (C7 : 0) FA, especially as C17 : 0 fatty acyl moieties can be metabolised to C7 : 0 moieties after five cycles of *β*-oxidation (online Supplementary Fig. S2). It is hypothesised that triheptanoin may influence anaplerotic mechanisms in the brain by replenishing tricarboxylic acid cycle substrates and intermediates and in effect improve and/or support mitochondrial metabolic pathways^(^ ^38^ ^,^ ^39^ ^)^. In a rat model of cortical spreading depression, it was shown that triheptanoin decreased brain cerebral excitability during short-term KD treatment^(^ ^40^ ^)^. Conversely, Costa *et al.* ^(41)^ showed that heptanoate is predominately metabolised by glia and proposed that the anticonvulsant effects of triheptanoin may in fact stem from glial metabolism. Although the exact mechanisms are currently unknown, a myriad of published literature have highlighted positive neurological effects of triheptanoin^(^ ^38^ ^,^ ^39^ ^,^ ^42^ ^–^ ^45^ ^)^.

As discussed, LCFA are unable to cross the mitochondrial membrane and require the carnitine shuttle system before being metabolised by *β*-oxidation in the mitochondrial matrix. One study investigating the effects of sunflower oil (LCT) consumption in healthy children showed that a general increase was seen in all straight-chain acylcarnitines^(^ ^46^ ^)^. It has been hypothesised that efflux of acylcarnitines occurs in healthy individuals as a mechanism of freeing the substrate CoA for other metabolic processes and that acylcarnitine efflux is thought to depend on intracellular acylcarnitine concentrations^(^ ^47^ ^)^. Alternatively, acylcarnitine metabolites detected in the blood may also originate from intestinal re-absorption of bile acylcarnitines^(^ ^47^ ^)^. We showed that three acylcarnitine metabolites, with acyl chains of C16 : 0, C18 : 1 and C18 : 0, were significantly lower during the MCTD phase. The placebo diet, presented in this study, was completely void of MCT but instead supplemented with lard, which contains LCT^(^ ^7^ ^)^. Higher abundances of acylcarnitine metabolites seen in the placebo diet may be attributed to increased acylcarnitine formation from the additional 5·5 % LCT present in the placebo diet, and subsequent exportation of these acylcarnitines out of cells and into the blood.

## *Conclusion*

Considering the consistent neuroprotective effectiveness seen in different MCT KD, it is likely that the overall mechanistic pathways involved are multifactorial. We showed that consumption of the MCTD by dogs with idiopathic epilepsy resulted in an increase in C17 : 0 fatty acyl chain containing metabolites in their serum, when compared with a standardised placebo diet. Future studies should aim to characterise the involvement and importance of C17 : 0 fatty acyl chain or odd-number fatty acyl chain moieties in MCT metabolism. Elucidating the exact mechanisms of MCT metabolic pathways and the mechanisms of action resulting in neuroprotective effects will undoubtedly facilitate the development of novel treatment strategies for both canines and humans with undesirable neurological conditions.

## Supplementary material

For supplementary material accompanying this paper visit http://dx.doi.org/10.1017/S0007114518001617. click here to view supplementary material
