# Supplementary material for: Metabolic perturbations associated with the consumption of a ketogenic medium-chain TAG diet in dogs with idiopathic epilepsy
Source: Br J Nutr. 2018 Jul 13;120(5):484–90. doi: 10.1017/S0007114518001617 (PMC6137430; doi:10.1017/S0007114518001617)
Supplement: Supplementary file 1 [file S0007114518001617sup001.docx]

| **Dog breed** | **Age**  **(years.months)** | **Weight**  **(kg)** | **Sex**  **(M/F)** | **Neuter status**  **(Y/N)** |
| --- | --- | --- | --- | --- |
| English Springer Spaniel | 5.5 | 21.60 | F | Y |
| Boxer | 6.2 | 30.15 | F | N |
| English Bull Terrier | 2.4 | 29.90 | F | Y |
| Cross breed | 2.2 | 25.90 | F | Y |
| Saint Bernard | 3.8 | 62.40 | F | N |
| Ihasn Aspo | 3.0 | 9.45 | F | Y |
| Golden retriever | 3.2 | 38.90 | M | N |
| Cross breed | 2.2 | 26.50 | M | Y |
| Rhodesian Ridgeback | 4.3 | 46.30 | M | N |
| Welsh Springer Spaniel | 4.3 | 25.15 | M | Y |
| Beagle | 6.8 | 14.95 | M | Y |
| American Bulldog | 4.3 | 33.80 | M | Y |
| Border Collie | 4.6 | 26.70 | M | N |
| Border Collie | 4.3 | 17.30 | M | Y |
| Cavalier King Charles | 4.7 | 10.20 | M | Y |
| Mastiff | 5.7 | 63.70 | M | Y |
| German Shepherd | 3.8 | 36.75 | M | N |
| Cross breed | 9.7 | 13.50 | M | Y |
| Siberian Huskey | 4.2 | 34.10 | M | Y |
| Beagle | 5.9 | 21.30 | M | N |
| Slovakian Rough Haired Pointer | 5.5 | 36.95 | M | Y |

**Supplementary Table 1.** Breed, age, weight, sex and neuter status of the all dogs included in this study.

| Serum Sample Collection |
| --- |
| All samples were collected by qualified personnel at the Royal Veterinary College Queen Mother Hospital for animals, processed and stored within one hour of sample collection. Canine blood samples were collected by needle (via cephalic, saphenous, femoral or jugular veins) into BD vacutainer (no additive) tubes. Extracted serum was kept frozen, transported at -80^o^C, and thawed for 30 minutes on ice prior to sample preparation. |
| Serum Sample Preparation |
| 100μl of canine serum was combined with 300μl of pre-chilled (-20ºC) methanol (Chromasolv, LC-MS grade, Fluka, Germany) in new plastic Eppendorf tubes, vortex mixed for 30 seconds and left to incubate at -20ºC for 4 hours. Samples were then left on ice for 20 minutes, after which samples were centrifuged at 13,000 rpm at 4ºC for 10 minutes. The supernatant was split into 2 sets of new plastic Eppendorf tubes with each tube containing 195μl of serum/methanol (1:3). All tubes were left to dry for 4 hours in a centrifugal evaporator (SpeedVac, Eppendorf, Germany) at 45ºC with lids left open. Dried metabolite extracts were re-suspended in 80μl of UPLC-grade water for RP-UPLC-MS and in 80μl of methanol/water (1:1) for LP-UPLC-MS. All samples were thoroughly vortex mixed and sonicated at room temperature to ensure dried metabolites were sufficiently re-suspended. 70μl of each sample, including the pooled quality control (QC) sample, was transferred into 96 well plates prior to analysis. |

**Supplementary Table 2.** Canine serum sample collection and preparation protocol.

| Reversed Phase Ultra Performance Liquid Chromatography | | | | | |
| --- | --- | --- | --- | --- | --- |
| Chromatography column: | | | Acquity UPLC C_18_ HSS (High Strength Silica) column (2.1mm x 100mm, 1.8μm particles; Waters Corporation, USA) | | |
| Column temperatures: | | | 40 ^o^C | | |
|  |  | (H_2_O, 0.1% FA) | | (MeOH, 0.1% FA) |  |
| Stage | Time (mins) | Mobile Phase A (%) | | Mobile Phase B (%) | Flow rate (mL/min) |
| Initial | 0.00 | 99.9 | | 0.1 | 0.4 |
| 1 | 2.00 | 99.9 | | 0.1 | 0.4 |
| 2 | 6.00 | 75.0 | | 25.0 | 0.4 |
| 3 | 10.00 | 20.0 | | 80.0 | 0.4 |
| 4 | 12.00 | 10.0 | | 90.0 | 0.4 |
| 5 | 21.00 | 0.1 | | 99.9 | 0.4 |
| 6 | 21.00 | 0.1 | | 99.9 | 0.6 |
| 7 | 25.00 | 0.1 | | 99.9 | 0.6 |
| 8 | 25.01 | 99.9 | | 0.1 | 0.4 |
| 9 | 28.00 | 99.9 | | 0.1 | 0.4 |
| Lipid Profiling Ultra Performance Liquid Chromatography | | | | | |
| Chromatography column: | | | Acquity UPLC C_18_ CSH (Charged Surface Hybrid) column (2.1mm x 100mm, 1.7μm particles; Waters Corporation, USA) | | |
| Column temperatures: | | | 55^o^C | | |
|  |  | (ACN:H_2_O (v/v) (60:40), 0.1% (v/v) FA, 10mM ammonium formate) | | (IPA:ACN (90:10), 0.1% (v/v) FA, 10mM ammonium formate) |  |
| Stage | Time (mins) | Mobile Phase A / (%) | | Mobile Phase B / (%) | Flow rate (mL/min) |
| Initial | 0.00 | 60 | | 40 | 0.5 |
| 1 | 2.00 | 57 | | 43 | 0.5 |
| 2 | 2.10 | 50 | | 50 | 0.5 |
| 3 | 12.00 | 46 | | 54 | 0.5 |
| 4 | 12.10 | 30 | | 70 | 0.5 |
| 5 | 18.00 | 1 | | 99 | 0.5 |
| 6 | 18.10 | 60 | | 40 | 0.5 |
| 7 | 20.00 | 60 | | 40 | 0.5 |

**Supplementary Table 3**. Gradient program for chromatography of reversed phase (RP-) and lipid profiling (LP-) UPLC-MS methodologies. Injection volumes of 4μl and 5μl were used in RP-UPLC and LP-UPLC respectively. All reagents/solvents were LC-MS grade. Key; Stage, distinctive stage of gradient change initiation; Time, time during analytical run; RP, reversed phase; LP, lipid profiling; H_2_O, water (Fisher Scientific); FA, formic acid (Sigma, USA); MeOH, methanol (Sigma, USA); ACN, Acetonitrile (Honeywell); IPA, Isopropanol (Honeywell).

| Mass Spectrometer Parameters in UPLC-MS Experiments | | |
| --- | --- | --- |
| Mass Spectrometer | Xevo G2-S Q-TOF | Q-TOF Premier |
| ESI (+) Capillary Voltage [kV] | 1.5 | 3.0 |
| ESI (-) Capillary Voltage [kV] | 1.2 | 2.5 |
| ESI (+) Sample Cone Voltage [V] | 20 | 30 |
| ESI (-) Sample Cone Voltage [V] | 30 | 25 |
| Source Temperature [^o^C] | 120 | 120 |
| Desolvation Temperature [ ^o^C] | 350 | 400 |
| Cone Gas Flow [L/hour] | 50.0 | 25 |
| Desolvation gas flow [L/hour] | 600.0 | 800 |
| Scan Range [Da] | 50-1200 | 50-1200 |
| Scan Duration [sec] | 0.2 | 0.3 |

**Supplementary Table 4**. Mass analysers and parameters used for mass spectrometric analysis. Using the LockSpray interface (Waters Corporation) leucine encephaline (m/z of 556.277 (ESI+), 554.262 (ESI-)), (200pg/μl in 50:50 (v/v) CH_3_CN:H_2_O, 0.1% (v/v) FA) was infused at 20μl/min as the lock mass reference to optimise instrument mass accuracy. UPLC-MS/MS data were collected with collision energy at 10V and 50V in reversed phase UPLC-MS and at 10V and 40V in lipid profiling UPLC-MS. Key; kV, kilovolts; V, volts; ^o^C, degree Celsius; L, liters; Da, Daltons; sec, seconds.

| XCMS Spectral Data Processing Parameters |
| --- |
| Library(xcms)  Loads XCMS package into workspace |
| <xcmsSet(method="centWave", peakwidth=c(5,20), ppm=15, snthresh=5)  XCMS centWave function parameters include; peakwidth, defines the accepted peak width range; ppm, defines the mass spectrometer accuracy; snthresh, defines order of magnitude above background noise peaks need to be to be accepted; centWave, defines the method of peak picking |
| <group(X, method="density", mzwid=0.03, bw=3)  XCMS group density function parameters include; mzwid, defines width of overlapping m/z slices to use for creating peak density chromatograms and grouping peaks across samples; bw, defines bandwidth |
| <rector(X, method="obiwarp", profstep=1)  The rector obiwarp function parameters include; profstep, defines step size (in m/z) to use for profile generation from raw data files |
| <-fillPeaks(X,method="chrom")  Peak groups that are missing peaks in some samples can be filled by reading the raw data files and integrating peaks in the regions of missing peaks |
| <Reorttab<-diffreport(X, "group1", "group2", "group1_group2")  Function facilitates the export of metabolite features with mass and retention time information in tab separated values (TSV) format which was imported into excel for further data processing. |

**Supplementary Table 5**. XCMS spectral data processing protocol and parameters. MS chromatograms and spectra were visualised using MassLynx software (version 4.1, Waters Corporation). Raw data files generated by UPLC-MS experiments were converted to NetCDF for further processing using Databridge software implemented in MassLynx software. Further spectral processing was carried out using R programming language (open-source software) and XCMS software package. Scripts used for spectral pre-processing and parameters are summarised.

| **Metabolite** | **Molecular** | **p** | **p(FDR)** | **Retention** | **Mass m/z** | **Mass m/z** | **Δppm** |
| --- | --- | --- | --- | --- | --- | --- | --- |
| **Name** | **Formula** | **[M v P]** | **[M v P]** | **Time (Sec)** | **(Detected)** | **(Theoretical)** |  |
| **Lipid Profiling UPLC-MS detected metabolic features identified based on the accurate [M+H]^+^ adduct mass** | | | | | | | |
| Creatinine | (C4H7N3O) | 0.0058 | 0.0432 | 33.5000 | 114.0655 | 114.0662 | 6 |
| C14:1 Acylcarnitine | (C21H39NO4) | 0.0058 | 0.0432 | 50.5000 | 370.2958 | 370.2952 | 2 |
| C16:1 Acylcarnitine | (C23H43NO4) | 0.0069 | 0.0467 | 60.2000 | 398.3273 | 398.3265 | 2 |
| C18:2 Acylcarnitine | (C25H45NO4) | 0.0050 | 0.0393 | 64.5000 | 424.3430 | 424.3421 | 2 |
| LysoPE(18:2) | (C23H44NO7P) | 0.0028 | 0.0262 | 66.0000 | 478.2940 | 478.2928 | 3 |
| (C30H46O7) | (C30H46O7) | 0.0012 | 0.0135 | 73.9000 | 519.3293 | 519.3316 | 4 |
| TG(46:5) | (C49H84O6) | 0.0060 | 0.0438 | 869.8000 | 769.6364 | 769.6341 | 3 |
| PC(36:5) | (C44H78NO8P) | 0.0008 | 0.0103 | 370.9000 | 780.5561 | 780.5538 | 3 |
| PA(42:2) | (C45H85O8P) | 0.0012 | 0.0137 | 869.8000 | 785.6098 | 785.6055 | 6 |
| TG(48:6) | (C51H86O6) | 0.0031 | 0.0274 | 871.1000 | 795.6528 | 795.6497 | 4 |
| PC(38:7) | (C46H78NO8P) | 0.0031 | 0.0274 | 352.5000 | 804.5554 | 804.5538 | 2 |
| PC(38:7) | (C46H78NO8P) | 0.0062 | 0.0443 | 268.5000 | 804.5586 | 804.5538 | 6 |
| PC(38:5) | (C46H82NO8P) | 0.0003 | 0.0049 | 489.1000 | 808.5863 | 808.5851 | 2 |
| PC(38:4) | (C46H84NO8P) | 0.0067 | 0.0456 | 389.5000 | 810.5979 | 810.6007 | 3 |
| PC(38:4) | (C46H84NO8P) | 0.0043 | 0.0351 | 432.6000 | 810.6041 | 810.6007 | 4 |
| PS(38:3) | (C44H80NO10P) | 0.0023 | 0.0231 | 234.4000 | 814.5657 | 814.5593 | 8 |
| PC(40:5) | (C48H86NO7P) | 0.0005 | 0.0063 | 431.4000 | 820.6265 | 820.6215 | 6 |
| PE(42:5) | (C47H84NO8P) | 0.0000 | 0.0001 | 413.1000 | 822.6053 | 822.6007 | 6 |
| PC(40:7) | (C48H82NO8P) | 0.0033 | 0.0280 | 339.1000 | 832.5892 | 832.5851 | 5 |
| PC(40:7) | (C48H82NO8P) | 0.0007 | 0.0092 | 325.3000 | 832.5901 | 832.5851 | 6 |
| PC(40:6) | (C48H84NO8P) | 0.0000 | 0.0001 | 366.3000 | 834.6087 | 834.6007 | 10 |
| PI(35:2) | (C44H81O13P) | 0.0067 | 0.0456 | 372.8000 | 849.5520 | 849.5488 | 4 |
| TG(52:5) | (C55H96O6) | 0.0002 | 0.0046 | 928.3000 | 853.7311 | 853.7280 | 4 |
| TG(52:4) | (C55H98O6) | 0.0004 | 0.0061 | 945.0000 | 855.7472 | 855.7436 | 4 |
| PC(42:8) | (C50H84NO8P) | 0.0001 | 0.0016 | 474.6000 | 858.6058 | 858.6007 | 6 |
| PI(37:4) | (C46H81O13P) | 0.0066 | 0.0456 | 352.3000 | 873.5530 | 873.5488 | 5 |
| TG(54:6) | (C57H98O6) | 0.0063 | 0.0446 | 930.2000 | 879.7476 | 879.7436 | 5 |
| TG(54:5) | (C57H100O6) | 0.0038 | 0.0317 | 945.7000 | 881.7630 | 881.7593 | 4 |
| PI(39:4) | (C48H85O13P) | 0.0033 | 0.0280 | 474.6000 | 901.5833 | 901.5801 | 4 |
| TG(58:10) | (C61H98O6) | 0.0010 | 0.0118 | 908.5000 | 927.7490 | 927.7436 | 6 |
| **Metabolic features with one or more potential metabolite identification for [M+H]+ adduct mass (Lipid Profiling)** | | | | | | | |
| MGDG(36:2) | (C45H82O10) | 0.0052 | 0.0403 | 848.8000 | 783.5928 | 783.5898 | 4 |
| PA(42:3) | ^ | ^ | ^ | ^ | ^ | ^ | ^ |
| PC(37:4) | (C45H82NO8P) | 0.0000 | 0.0011 | 407.9000 | 796.5877 | 796.5851 | 3 |
| PE(40:4) | ^ | ^ | ^ | ^ | ^ | ^ | ^ |
| (18:2)-Glc-cholesterol | (C51H86O7) | 0.0004 | 0.0058 | 604.0000 | 811.6518 | 811.6446 | 9 |
| (16:2)-Glc-Sitosterol | ^ | ^ | ^ | ^ | ^ | ^ | ^ |
| (16:1)-Glc-Stigmasterol | ^ | ^ | ^ | ^ | ^ | ^ | ^ |
| PE(42:4) | (C47H86NO8P) | 0.0000 | 0.0001 | 540.7000 | 824.6218 | 824.6164 | 7 |
| PC(39:4) | ^ | ^ | ^ | ^ | ^ | ^ | ^ |
| PE(43:4) | (C48H88NO8P) | 0.0052 | 0.0403 | 572.1000 | 838.6376 | 838.6320 | 7 |
| PC(40:4) | ^ | ^ | ^ | ^ | ^ | ^ | ^ |
| **Metabolic features which were not identified based on [M+H]+ adduct mass (Lipid Profiling)** | | | | | | | |
| UNKNOWN | N/A | 0.0000 | 0.0002 | 430.7000 | 822.6226 | N/A | N/A |
| UNKNOWN | N/A | 0.0071 | 0.0472 | 945.7000 | 824.7748 | N/A | N/A |
| UNKNOWN | N/A | 0.0021 | 0.0221 | 463.2000 | 833.5941 | N/A | N/A |
| UNKNOWN | N/A | 0.0075 | 0.0486 | 911.9000 | 852.7189 | N/A | N/A |
| UNKNOWN | N/A | 0.0003 | 0.0050 | 490.4000 | 876.5786 | N/A | N/A |
| UNKNOWN | N/A | 0.0072 | 0.0472 | 913.9000 | 893.7031 | N/A | N/A |
| UNKNOWN | N/A | 0.0000 | 0.0002 | 370.2000 | 1159.3323 | N/A | N/A |
| UNKNOWN | N/A | 0.0000 | 0.0003 | 370.2000 | 1159.8340 | N/A | N/A |
| UNKNOWN | N/A | 0.0062 | 0.0443 | 353.8000 | 1196.3348 | N/A | N/A |
| **Metabolic features identified based on the accurate [M+FA-H]^-^ adduct mass** | | | | | | | |
| acyclic olefins | C7H14 | 0.0008 | 0.0186 | 580.7 | 143.1076 | 143.1078 | 1 |
| Unsaturated Heptanoic acid | C7H12O2 | 0.0003 | 0.0124 | 482.1 | 173.0820 | 173.0819 | 0 |
| p-Toluenesulfonic acid | C7H8O3S | 0.0028 | 0.0459 | 295.6 | 217.0177 | 217.0119 | 0 |
| Heptadecanal | C17H34O | 0.0032 | 0.0496 | 766.0 | 299.2591 | 299.2592 | 0 |
| Docosatrienoic acid | C22H38O2 | 0.0002 | 0.0103 | 917.8 | 379.2827 | 379.2854 | 7 |
| fatty acyl glycoside | C19H30O7 | 0.0005 | 0.0146 | 593.6 | 415.1970 | 415.1974 | 1 |
| LysoPE(22:0) | C27H56NO7P | 0.0029 | 0.0479 | 815.2 | 582.3779 | 582.3776 | 0 |
| endo-1,4-beta-Xylanase | C34H50N4O2 | 0.0008 | 0.0186 | 920.3 | 591.3912 | 591.3916 | 1 |
| triterpenoids | C34H50O7 | 0.0002 | 0.0103 | 744.1 | 615.3493 | 615.3539 | 7 |
| SM(36:1) | C41H83N2O6P | 0.0000 | 0.0023 | 1139.0 | 775.5964 | 775.5971 | 1 |
| PC(34:2) | C42H80NO7P | 0.0008 | 0.0186 | 1151.7 | 786.5661 | 786.5654 | 1 |
| PC(40:4) | C48H88NO8P | 0.0012 | 0.0222 | 1199.8 | 882.6143 | 882.6230 | 10 |
| **Metabolic features with one or more potential metabolite identification for [M+FA-H]- adduct mass** | | | | | | | |
| Drospirenone | C24H30O3 | 0.0001 | 0.0041 | 918.0 | 411.2209 | 411.2177 | 8 |
| Umbelliprenin | ^ | ^ | ^ | ^ | ^ | ^ | ^ |
| LysoPC(20:1) | C28H56NO7P | 0.0002 | 0.0103 | 800.9 | 594.3776 | 594.3776 | 0 |
| PC(18:1/2:0) | ^ | ^ | ^ | ^ | ^ | ^ | ^ |
| Glucosylceramide (34:1) | C40H77NO8 | 0.0008 | 0.0186 | 1049.7 | 744.5644 | 744.5631 | 2 |
| Galactosylceramide (34:1) | ^ | ^ | ^ | ^ | ^ | ^ | ^ |
| PC(36:1) | C44H86NO8P | 0.0002 | 0.0118 | 1190.3 | 832.5980 | 832.6073 | 11 |
| PE(39:1) | ^ | ^ | ^ | ^ | ^ | ^ | ^ |
| **Metabolic features which were not identified based on [M+FA-H]- adduct mass** | | | | | | | |
| UNKNOWN | N/A | 0.0008 | 0.0186 | 848.7 | 283.2641 | N/A | N/A |
| UNKNOWN | N/A | 0.0031 | 0.0496 | 836.1 | 307.2642 | N/A | N/A |
| UNKNOWN | N/A | 0.0004 | 0.0132 | 593.6 | 437.1795 | N/A | N/A |
| UNKNOWN | N/A | 0.0000 | 0.0004 | 918.2 | 440.2246 | N/A | N/A |
| UNKNOWN | N/A | 0.0013 | 0.0245 | 816.5 | 610.3906 | N/A | N/A |
| UNKNOWN | N/A | 0.0012 | 0.0222 | 768.0 | 622.3346 | N/A | N/A |
| UNKNOWN | N/A | 0.0000 | 0.0004 | 862.4 | 663.4719 | N/A | N/A |
| UNKNOWN | N/A | 0.0008 | 0.0186 | 743.9 | 682.3337 | N/A | N/A |
| UNKNOWN | N/A | 0.0004 | 0.0132 | 780.2 | 727.4611 | N/A | N/A |
| UNKNOWN | N/A | 0.0010 | 0.0203 | 1082.3 | 918.5485 | N/A | N/A |
| UNKNOWN | N/A | 0.0005 | 0.0158 | 1200.4 | 948.5947 | N/A | N/A |

**Supplementary Table 6**: List of metabolites shown to be significantly different in abundance between the placebo diet phase and MCTD phase. Metabolites were detected in positive (+) electrospray ionisation (ESI) mode using lipid profiling (LP) liquid chromatography (LC) coupled to a Q-TOF mass spectrometer and in in negative (-) electrospray ionisation (ESI) mode using reversed phase (RP) liquid chromatography (LC) coupled to a Q-TOF mass spectrometer. Significant metabolites were determined using paired student t-test with Benjamini Hochberg FDR adjustment, (p<0.05) and identified in UPLC-MS/MS experiments. Metabolites highlighted in grey were higher in abundance during the placebo diet phase whereas metabolites that are not highlighted were higher in abundance during the MCTD phase. Key: M, MCTD phase; P, Placebo diet phase; Δppm, deviation between measured mass and theoretical mass in ppm calculated; CV(%), coefficient of variation calculated based on total number of pooled QC samples acquired during experimental run.

**
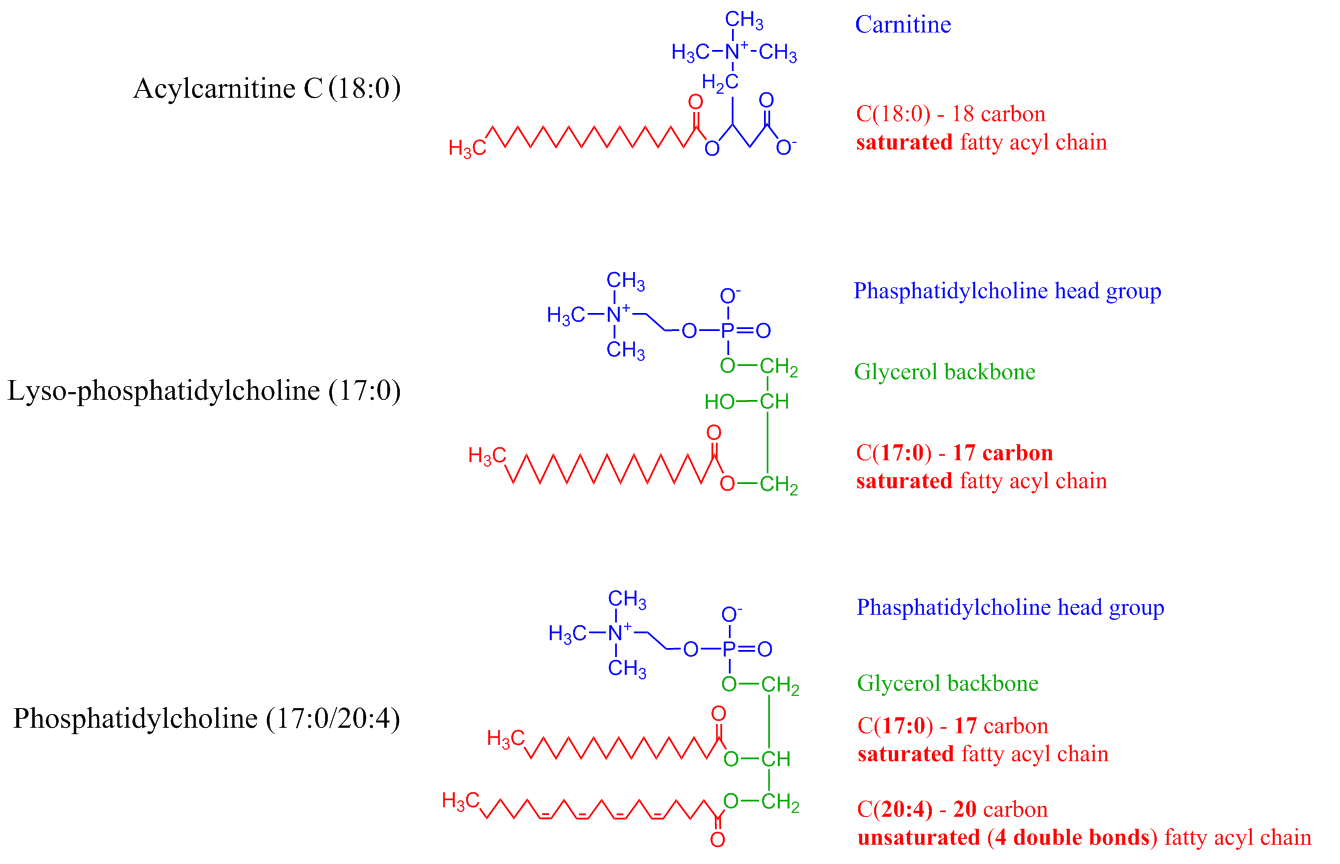
**

**Supplementary Figure 1**: Structure of Acylcarnitine C(18:0), LysoPC(17:0) and PC(17:0/20:4). Fatty acyl moieties are represented by the number of carbons followed by number of double bonds on each fatty acyl chain, where (20:4) represents a 20 carbon, unsaturated fatty acyl moiety containing 4 double bonds. Key; LysoPC, Lyso-phosphatidylcholine; PC, Phosphatidylcholine.

**
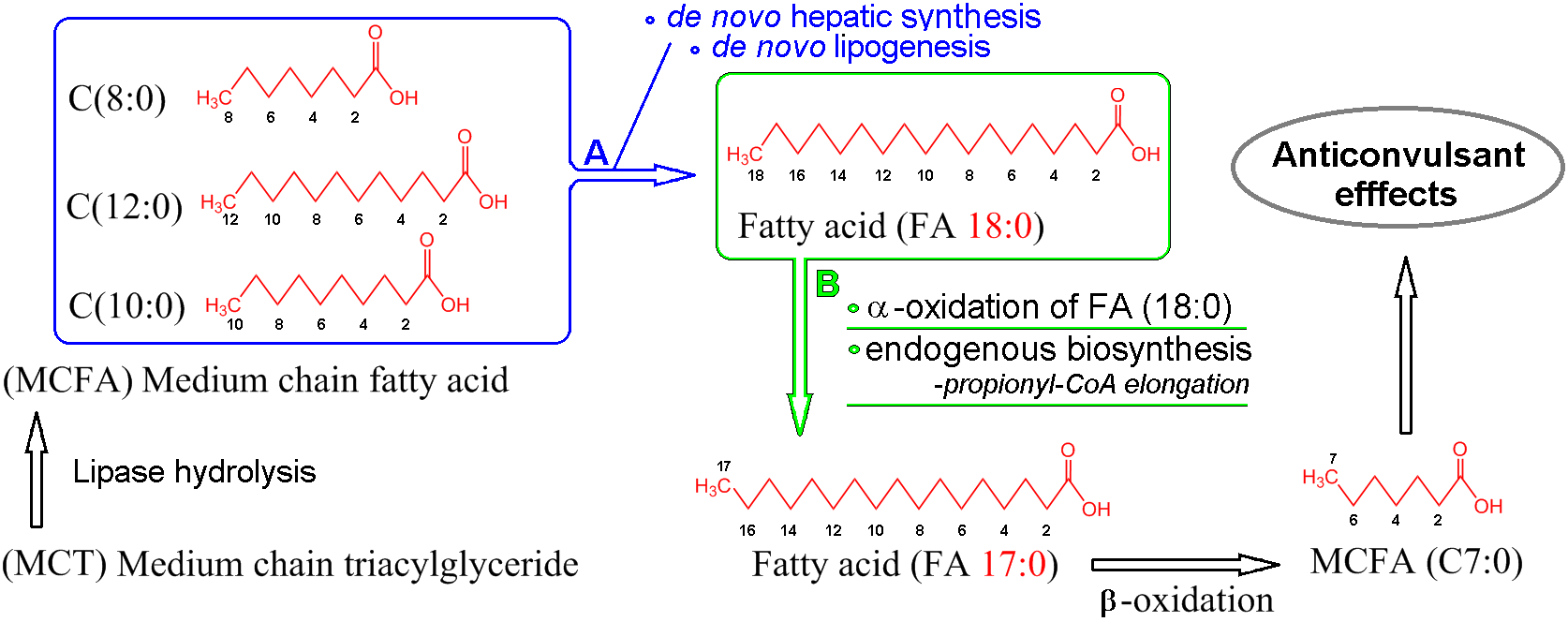
**

**Supplementary Figure 2**: Schematic illustration of proposed metabolic pathways for (A) *de novo* hepatic synthesis and/or *de novo* lipogenesis from medium chain fatty acids and (B) biosynthesis of 17 carbon fatty acyl moieties by way of α-oxidation and/or endogenous biosynthesis. Key: MCT, medium chain triacylglyceride; MCFA medium chain fatty acids; FA (17:0), Fatty acid containing 17 carbon saturated fatty acyl moiety.

**
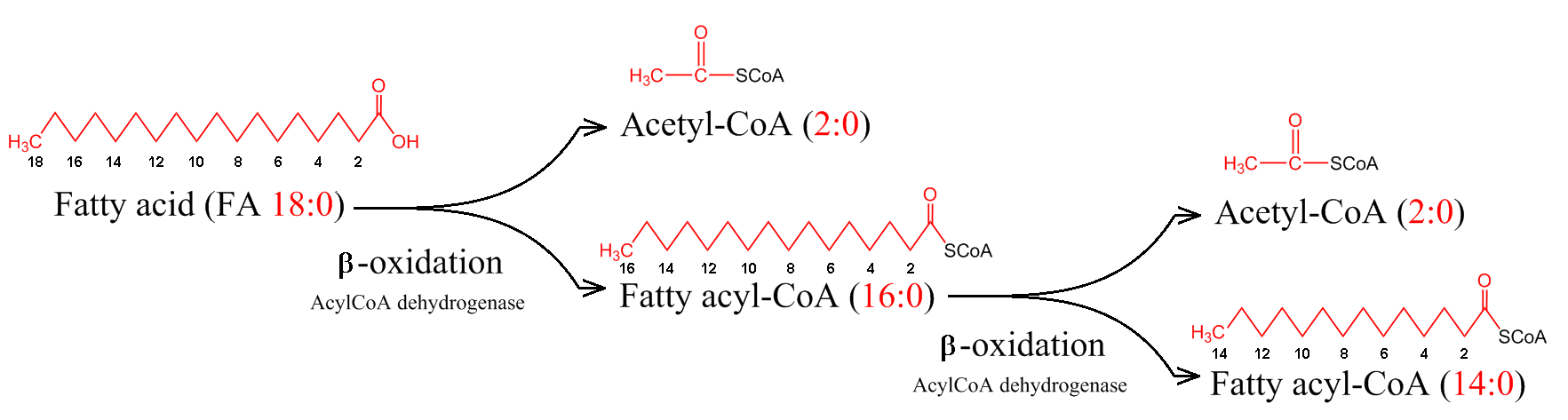
**

**Supplementary Figure 3**: Illustration demonstrating the 2 carbon cleavage of fatty acids during β-oxidation fatty acid metabolism.
